# The effect of VTA stimulation on hippocampal beta amyloid, tau protein accumulation and oxidative stress

**DOI:** 10.1002/alz.087613

**Published:** 2025-01-03

**Authors:** Fatemeh Bakhtiarzadeh, Javad Mir Najafi_Zadeh, Koorosh Shahpasand

**Affiliations:** ^1^ Tarbiat Modares university, Tehran Iran (Islamic Republic of); ^2^ Brain and Cognitive Sciences, Cell Science Research Center, Royan Institute for Stem Cell Biology & Technology, ACECR, Tehran, NY Iran (Islamic Republic of)

## Abstract

**Background:**

Alzheimer’s disease (AD) is a degenerative condition characterized by a progressive decline in cognitive function, predominantly affecting older individuals. AD is associated with a range of histopathological alterations, including the gradual demise of neuronal cells, the accumulation of amyloid plaques, and the formation of neurofibrillary tangles. Furthermore, research suggests that the brain tissue of AD patients is subject to oxidative stress, which manifests as the oxidation of proteins, lipids, DNA, and the process of glycoxidation, throughout the disease progression. Considering the potential effect of deep brain stimulation in the treatment of Alzheimer’s disease and the possible role of dopamine in this disease, in the present study the effect of the ventral tegmental area (VTA) stimulation was investigated in a mice model of Alzheimer‐like disease.

**Method:**

This Alzheimer‐like model was created by bilateral injecting cis‐phospho‐tau into the dorsal hippocampus. A bipolar electrode was implanted in the VTA for stimulation at the frequency of 100 Hz. In Alzheimer‐like animals, the effect of electrical stimulation on the amount of tau protein and beta amyloid accumulation by immunohistochemistry and oxidative stress by ELISA test were investigated.

**Result:**

The injection of cis‐phospho‐tau increased the phospho‐tau protein and beta‐amyloid accumulation. Applying VTA stimulation in Alzheimer‐like animals reduced tau and beta‐amyloid accumulation. In addition, it reduced some oxidative stress and inflammation indices, which were increased after the injection of cis‐phospho‐tau and increase anti‐oxidants.

**Conclusion:**

It seems that VTA stimulation reduce the accumulation of phosphor‐tau protein and amyloid beta, in addition factors involved in oxidative stress and inflammation. These ameliorative effects are at least partly through the activity of D1‐like dopamine receptors.

